# Supplementary material for: TMEM79/MATTRIN defines a pathway for Frizzled regulation and is required for Xenopus embryogenesis
Source: eLife. 2020 Sep 14;9:e56793. doi: 10.7554/eLife.56793 (PMC7521923; doi:10.7554/eLife.56793)
Supplement: Supplementary file 1. — Number of embryos examined for anterior phenotypes in Figure 6D. Supplementary File 1B. Number of embryos examined for pigmentation phenotype in Figure 6D. Supplementary File 1C. Number of embryos examined for neural versus epidermal marker expression in Figure 6F. Supplementary File 1D. Number of embryos examined for spina bifida in Figure 7B. Supplementary File 1E. Number of embryos examined for axial elongation defect in Figure 7D. Supplementary File 1F. Number of animal pole explants examined for axial mesoderm elongation in Figure 7F. Supplementary File 1G. Number of embryos examined for enlarged head or spina bifida in Figure 6—figure supplement 1B. Supplementary File 1H. Number of embryos examined for anterior marker expression in Figure 6—figure supplement 1G. Supplementary File 1I. Number of embryos examined for anterior phenotypes in Figure 6—figure supplement 1F. Supplementary File 1J. Number of embryos examined for dorsal/organizer marker expression in Figure 6—figure supplement 1G. Supplementary File 1K. Number of embryos examined for neural crest marker expression in Figure 6—figure supplement 1H. [file elife-56793-supp1.docx]

**Supplementary file 1**

**Supplementary File 1A. Number of embryos examined for anterior phenotypes in Figure 6D.**

|  | n | % of embryos | | | |
| --- | --- | --- | --- | --- | --- |
|  |  | Normal | Enlarged Head | Moderate Deficiency | Severe Deficiency |
| CoMO | 42 | 93.7 | 6.3 | 0.0 | 0.0 |
| Tmem79 MO | 27 | 22.1 | 50.4 | 27.5 | 0.0 |
| Tmem79 MO+TMEM79 | 29 | 68.2 | 9.5 | 22.3 | 0.0 |
| Tmem79 MO+Usp8 MO | 36 | 55.2 | 29.7 | 4.1 | 11.0 |
| Tmem79MO+β-catenin MO | 31 | 52.1 | 28.4 | 2.4 | 17.1 |

Moderate Deficiency: embryos with anterior structures reduced, such as small eyes and a small cement gland.  Severe Deficiency: embryos with no visible anterior structures, lacking eyes and the cement gland.

Supplementary File 1B. Number of embryos examined for pigmentation phenotype in Figure 6D.

|  | n | % of embryos | |
| --- | --- | --- | --- |
|  |  | Normal | Pigmentation Deficiency |
| Co MO | 42 | 97.6 | 2.4 |
| Tmem79 MO | 27 | 3.7 | 96.3 |
| Tmem79 MO+TMEM79 | 29 | 89.6 | 10.4 |
| Tmem79 MO+Usp8 MO | 36 | 72.2 | 27.8 |
| Tmem79MO+β-catenin MO | 31 | 74.2 | 25.8 |

**Supplementary File 1C. Number of embryos examined for neural versus epidermal marker expression in Figure 6F.**

|  | n | Sox2 | | n | Keratin | |
| --- | --- | --- | --- | --- | --- | --- |
|  |  | % of embryos | |  | % of embryos | |
|  |  | Normal | Reduced |  | Normal | Expanded |
| Co MO | 34 | 85.7 | 14.3 | 49 | 89.5 | 10.5 |
| Tmem79 MO | 42 | 31.8 | 68.2 | 58 | 38.9 | 61.1 |
| Tmem79 MO+Tmem79 | 53 | 73.9 | 26.1 | 45 | 76.0 | 24.0 |
| Tmem79 MO+Usp8MO | 47 | 70.2 | 29.8 | 42 | 73.8 | 26.2 |

**Supplementary File 1D. Number of embryos examined for spina bifida in Figure 7B.**

|  | N | % of embryos | |
| --- | --- | --- | --- |
|  |  | Normal | Spina Bifida |
| Co MO | 66 | 95.3 | 4.7 |
| Tmem79 MO | 74 | 14.7 | 85.3 |
| Tmem79 MO+Tmem79 | 44 | 63.5 | 36.5 |

**Supplementary File 1E. Number of embryos examined for axial elongation defect in Figure 7D.**

|  | N | % of embryos | |
| --- | --- | --- | --- |
|  |  | Normal | Axial Elongation Defect |
| Co MO | 46 | 100 | 0.0 |
| Tmem79 MO | 64 | 31.3 | 68.7 |
| Tmem79 MO +Tmem79 | 58 | 72.4 | 27.6 |

**Supplementary File 1F. Number of animal pole explants examined for axial mesoderm elongation in Figure 7F.**

|  | n | Length | Width | Ratio |
| --- | --- | --- | --- | --- |
|  |  | Average | Average |  |
| Co MO | 20 | 123.4±11.9 | 123.3±11.6 | 1.00 |
| Co MO (+Act) | 21 | 229.0±19.7 | 75.1±10.7 | 3.05 |
| Tmem79 MO (+Act) | 21 | 155.3±19.3 | 110.3±15.2 | 1.41 |
| Tmem79 MO+Tmem79 (+Act) | 19 | 203.7±21.9 | 98.4±14.4 | 2.07 |
| Tmem79 MO+Usp8 MO (+Act) | 18 | 208.7±24.9 | 88.4±15.4 | 2.36 |

Length and width are in μm

**Supplementary File 1G. Number of embryos examined for enlarged head or spina bifida in Figure 6-figure supplement 1B.**

|  | N | % of embryos | | |
| --- | --- | --- | --- | --- |
|  |  | Normal | Enlarged Head | Spina Bifida |
| Uninjected | 76 | 97.6 | 0.0 | 2.4 |
| TMEM79 | 93 | 18.2 | 54.5 | 27.3 |

**Supplementary File 1H. Number of embryos examined for anterior marker expression in Figure 6-figure supplement 1G.**

|  |  | n | % of embryos | |
| --- | --- | --- | --- | --- |
|  |  |  | Normal | Reduced |
| XAG | Co MO | 35 | 91.4 | 8.6 |
|  | Tmem79 MO | 34 | 64.7 | 35.3 |
|  | Tmem79 MO+Tmem79 | 32 | 81.3 | 18.8 |
| Bf1 | Co MO | 51 | 88.2 | 11.8 |
|  | Tmem79 MO | 44 | 27.3 | 72.7 |
|  | Tmem79 MO+Tmem79 | 50 | 68.0 | 32.0 |
| En2 | Co MO | 35 | 95.4 | 4.6 |
|  | Tmem79 MO | 34 | 54.7 | 45.3 |
|  | Tmem79 MO+Tmem79 | 32 | 71.3 | 28.8 |
| Krox20 | Co MO | 45 | 91.1 | 8.9 |
|  | Tmem79 MO | 67 | 32.8 | 67.2 |
|  | Tmem79 MO+Tmem79 | 50 | 66.0 | 34.0 |
| n-tub | Co MO | 34 | 94.1 | 5.9 |
|  | Tmem79 MO | 38 | 52.6 | 47.4 |
|  | Tmem79 MO+Tmem79 | 36 | 83.3 | 16.7 |

**Supplementary File 1I. Number of embryos examined for anterior phenotypes in Figure 6-figure supplement 1F.**

|  | n | % of embryos | | | |
| --- | --- | --- | --- | --- | --- |
|  |  | Normal | Moderate Deficiency | Severe Deficiency | Enlarged Head |
| Co MO | 64 | 92.2 | 7.8 | 0.0 | 0.0 |
| Usp8 MO | 48 | 6.8 | 0.0 | 0.0 | 93.2 |
| β-catenin MO | 34 | 15.8 | 0.0 | 0.0 | 84.2 |

**Supplementary File 1J. Number of embryos examined for dorsal/organizer marker expression in Figure 6-figure supplement 1G.**

|  |  | n | % of embryos | |
| --- | --- | --- | --- | --- |
|  |  |  | Normal | Affected |
| Gsc | Co MO | 38 | 88.9 | 11.1 |
|  | Tmem79 MO | 39 | 89.5 | 10.5 |
|  | Tmem79 MO+Tmem79 | 38 | 88.9 | 11.1 |
| Chd | Co MO | 39 | 89.5 | 10.5 |
|  | Tmem79 MO | 42 | 81.8 | 18.2 |
|  | Tmem79 MO+Tmem79 | 37 | 82.4 | 17.6 |
| Dkk | Co MO | 36 | 100.0 | 0.0 |
|  | Tmem79 MO | 40 | 90.0 | 10.0 |
|  | Tmem79 MO+Tmem79 | 39 | 84.2 | 15.8 |
| Lim1 | Co MO | 36 | 100.0 | 0.0 |
|  | Tmem79 MO | 40 | 90.0 | 10.0 |
|  | Tmem79 MO+Tmem79 | 39 | 84.2 | 15.8 |
| Xnr3 | Co MO | 17 | 100.0 | 0.0 |
|  | Tmem79 MO | 20 | 90.0 | 10.0 |
|  | Tmem79 MO+Tmem79 | 16 | 87.5 | 12.5 |
| xNot | Co MO | 29 | 90.0 | 10.0 |
|  | Tmem79 MO | 40 | 85.0 | 15.0 |
|  | Tmem79 MO+Tmem79 | 39 | 78.9 | 21.1 |

**Supplementary File 1K. Number of embryos examined for neural crest marker expression in Figure 6-figure supplement 1H.**

|  |  | n | % of embryos | |
| --- | --- | --- | --- | --- |
|  |  |  | Normal | Reduced |
| FoxD3 | Co MO | 42 | 94.1 | 5.9 |
|  | Tmem79 MO | 39 | 42.8 | 57.2 |
|  | Tmem79 MO+Tmem79 | 37 | 85.3 | 14.8 |
| Snail1 | Co MO | 51 | 92.1 | 7.9 |
|  | Tmem79 MO | 48 | 35.8 | 64.2 |
|  | Tmem79 MO+Tmem79 | 45 | 78.3 | 21.8 |
